# Spontaneous Retrobulbar Hematoma During a Hypertensive Crisis Managed Without Surgical Decompression: A Case Report

**DOI:** 10.7759/cureus.116418

**Published:** 2026-09-17

**Authors:** Hala Ait Amar, Houda Safwate, Assala Darragi Arfawi, Kenza Bennouna, Mohamed Elbelhadji

**Affiliations:** 1 Ophthalmology, Mohammed VI University of Health Sciences, Casablanca, MAR; 2 Maxillofacial Surgery, Mohammed VI University of Health Sciences, Casablanca, MAR

**Keywords:** acute proptosis, computed tomography, hypertensive crisis, nonsurgical resolution, orbital compartment syndrome, retrobulbar hematoma, spontaneous orbital hemorrhage

## Abstract

Spontaneous retrobulbar hematoma is an uncommon cause of acute painful proptosis and may rapidly threaten vision through orbital compartment syndrome. We report a 55-year-old woman with severe uncontrolled hypertension who presented with sudden painful right proptosis, profound visual loss, marked ocular tension, and restricted ocular motility. Computed tomography demonstrated a 16 × 20 mm right intraconal retrobulbar hematoma, while magnetic resonance angiography excluded a major underlying vascular abnormality. Urgent orbital decompression was planned. During preoperative preparation, the patient received intravenous methylprednisolone and antihypertensive therapy. Shortly thereafter, visual acuity recovered to 10/10 (20/20), intraocular pressure decreased to 18 mmHg, proptosis regressed, and ocular motility normalized. Because this improvement was rapid and sustained, decompression was deferred under strict ophthalmic monitoring with immediate surgical capability available if deterioration occurred. Follow-up imaging demonstrated progressive hematoma regression and complete radiological resolution at four weeks, without residual visual deficit. Medical therapy may have contributed to the improvement, although the relative contribution of each component of treatment cannot be determined from this single case. This case emphasizes repeated objective reassessment while maintaining urgent decompression as the standard approach when visual compromise persists or progresses.

## Introduction

Orbital compartment syndrome (OCS) results from an acute rise in intraorbital pressure that compromises perfusion of the retina and optic nerve. Because irreversible visual loss can occur rapidly, OCS is primarily a clinical diagnosis, and urgent orbital decompression should not be delayed when persistent or progressive visual compromise is present [1,2]. Retrobulbar hemorrhage is a classic precipitating event, most often after orbital trauma, periocular surgery, or injection.

Nontraumatic orbital hemorrhage is substantially less common. Reported associations include orbital vascular malformations, increased venous pressure, coagulopathies, antithrombotic therapy, systemic disorders, and severe hypertension [3,4]. The clinical spectrum ranges from self-limited hemorrhage to rapidly progressive OCS requiring emergency decompression [3-5]. Distinguishing these trajectories may be difficult at presentation because proptosis, increased orbital tension, visual loss, and motility restriction can initially suggest vision-threatening OCS. Medical treatment may be administered during emergency management, but any subsequent improvement should be interpreted cautiously because the relative contribution of each therapeutic intervention may be difficult to determine.

We report a spontaneous right intraconal retrobulbar hematoma occurring during severe uncontrolled hypertension, with an initial presentation highly concerning for OCS. Urgent decompression was planned, while intravenous methylprednisolone and antihypertensive therapy were initiated during preoperative management; oral corticosteroid therapy was subsequently continued. Rapid and sustained objective improvement occurred before surgery, allowing decompression to be deferred under strict ophthalmic monitoring. This case highlights the diagnostic and therapeutic challenge posed by a severe initial presentation that improves during preparation for emergency decompression and emphasizes the importance of repeated objective reassessment without attributing recovery to a single component of medical treatment.

## Case presentation

A 55-year-old woman with known hypertension presented to the emergency department approximately one hour after the sudden onset of painful right-sided proptosis associated with a marked decrease in vision (Figure 1).

**Figure 1:**
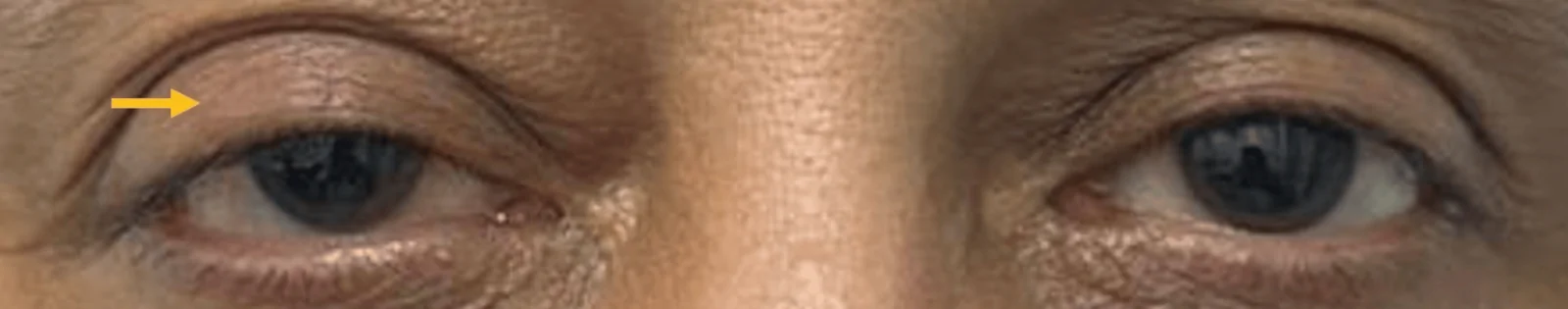
Clinical photograph at presentation showing right-sided ocular asymmetry The yellow arrow indicates the affected right eye. Objective radiological measurements of proptosis are provided on orbital computed tomography. Written informed consent was obtained from the patient for publication of this case report and accompanying images.

She had discontinued her antihypertensive treatment one week earlier. There was no history of ocular or craniofacial trauma, and she was not receiving antiplatelet or anticoagulant therapy. On admission, her blood pressure was 220/95 mmHg.

Best-corrected visual acuity was counting fingers in the right eye and 10/10 (20/20) in the left eye. Intraocular pressure in the right eye was initially unmeasurable because of extreme globe firmness, with a rock-hard globe on palpation; intraocular pressure in the left eye was 16 mmHg. The right eye was proptotic, without episcleral venous engorgement. Ocular motility showed limitation of elevation of the right eye. The cornea showed mild edema without significant loss of transparency, and the anterior chamber was quiet. There was no relative afferent pupillary defect. Color vision was assessed using the Lanthony desaturated D-15 hue test and was normal. Automated perimetry was not performed at presentation because the profound visual loss was considered to preclude reliable testing. Given the mild degree of corneal edema, preserved color vision, and absence of a relative afferent pupillary defect, the precise mechanism of the initial profound visual loss could not be definitively established. Mild corneal edema may have contributed to the visual impairment, while a transient pressure-related visual dysfunction was also considered.

Fundus examination and color fundus photography of the right eye demonstrated a small flame-shaped retinal hemorrhage superior to the optic disc, whereas the left fundus was unremarkable (Figure 2).

**Figure 2:**
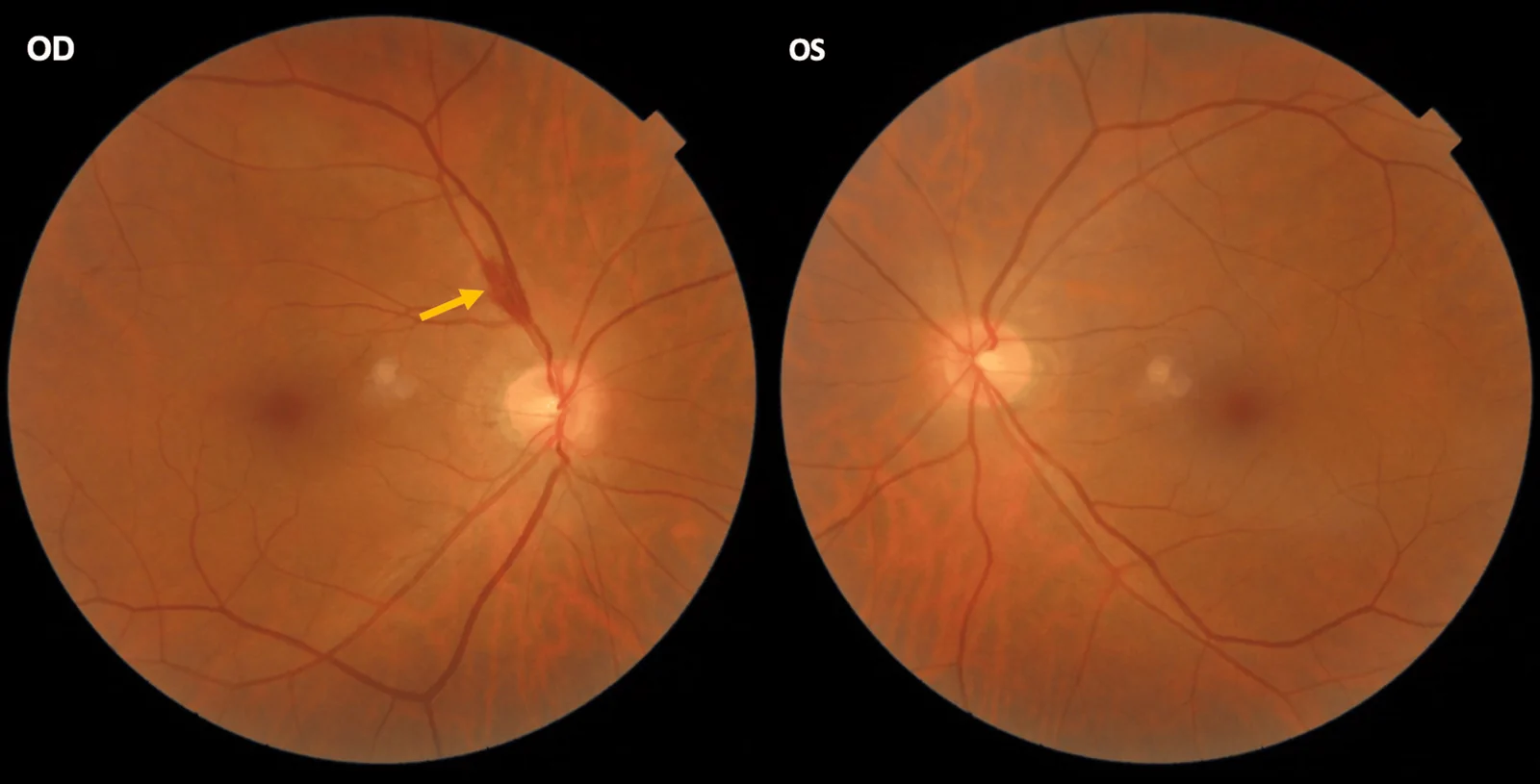
Color fundus photographs of both eyes at presentation The right eye (OD) shows a small flame-shaped retinal hemorrhage superior to the optic disc (yellow arrow), while the left eye (OS) is unremarkable.

Optical coherence tomography with ganglion cell analysis showed no significant structural abnormality in either eye (Figure 3).

**Figure 3:**
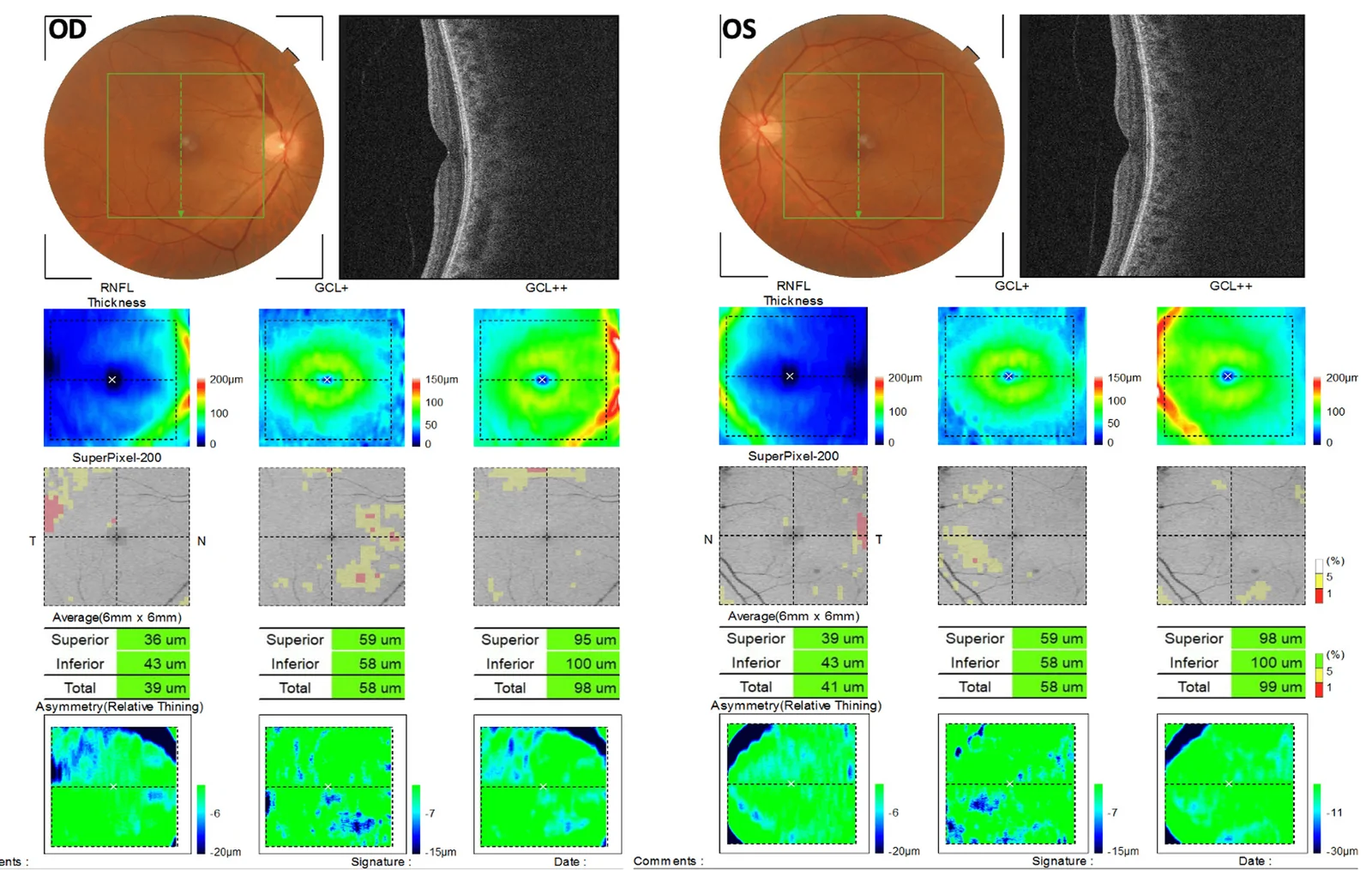
Optical coherence tomography with ganglion cell analysis showing no significant structural abnormality in either eye OD: right eye; OS: left eye; RNFL: retinal nerve fiber layer; GCL: ganglion cell layer

Complete blood count, including platelet count, as well as prothrombin time and activated partial thromboplastin time, were within normal limits, providing no laboratory evidence of thrombocytopenia or a coagulation disorder.

Given the combination of acute painful proptosis, profound visual loss, marked orbital tension with a rock-hard globe, and motility restriction, concern for OCS secondary to retrobulbar hemorrhage was high. Emergency orbital computed tomography (CT) demonstrated a 16 × 20 mm hyperdense right intraconal retrobulbar hemorrhagic collection compatible with a retrobulbar hematoma (Figure 4).

**Figure 4:**
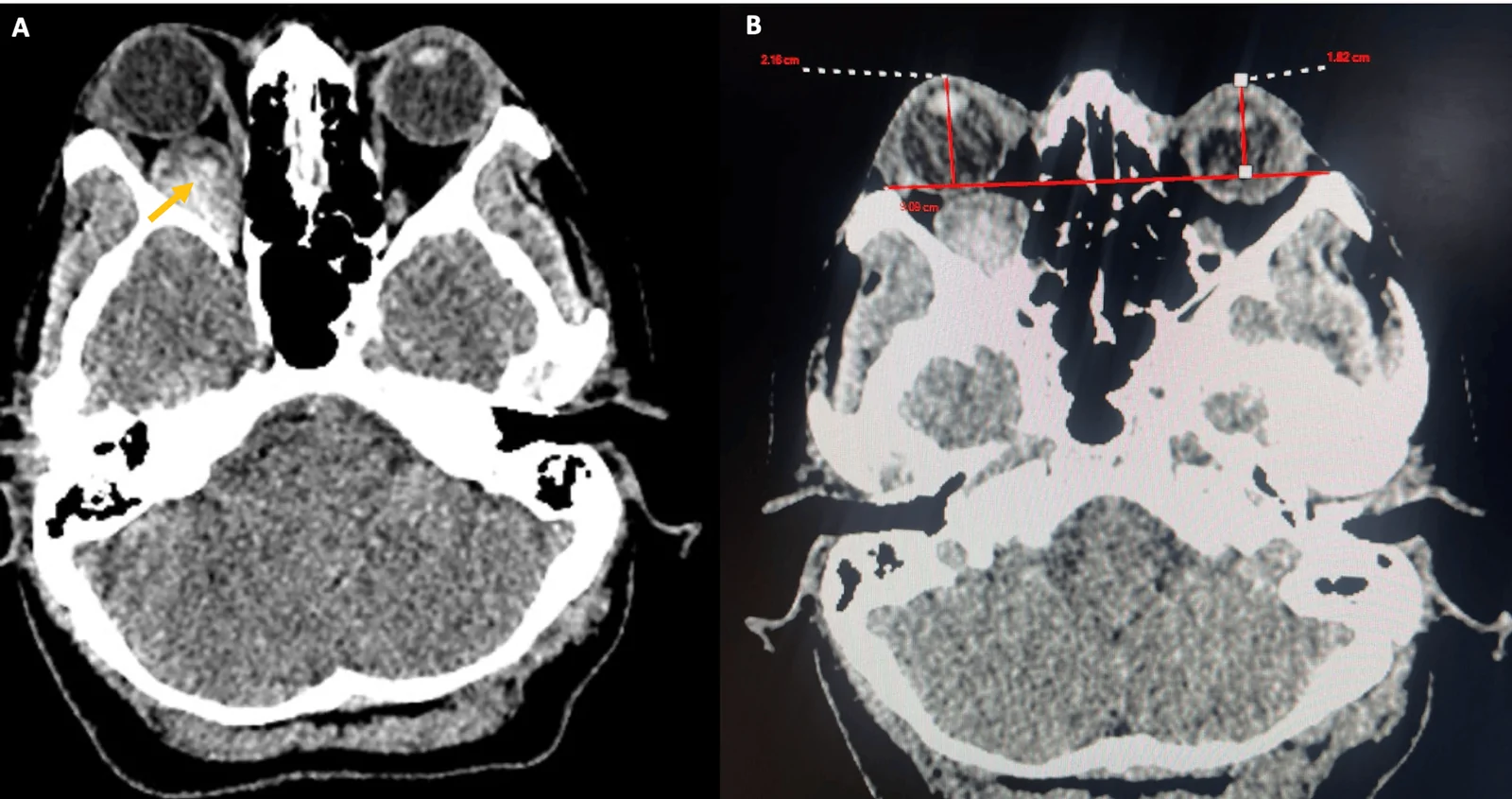
Initial orbital computed tomography showing right intraconal retrobulbar hematoma and radiological assessment of proptosis (A) Axial orbital computed tomography at presentation showing a hyperdense 16 × 20 mm right intraconal retrobulbar hematoma (yellow arrow). (B) CT-based assessment of globe position showing an anterior globe projection of 21.8 mm in the right eye and 18.2 mm in the left eye, corresponding to an interocular asymmetry of 3.6 mm and supporting the presence of right-sided proptosis.

CT-based assessment of globe position showed an anterior globe projection of 21.8 mm in the right eye and 18.2 mm in the left eye, corresponding to an interocular asymmetry of 3.6 mm and supporting the presence of right-sided proptosis.

Orbital magnetic resonance imaging (MRI) confirmed the 16 × 20 mm retrobulbar hemorrhagic collection (Figure 5).

**Figure 5:**
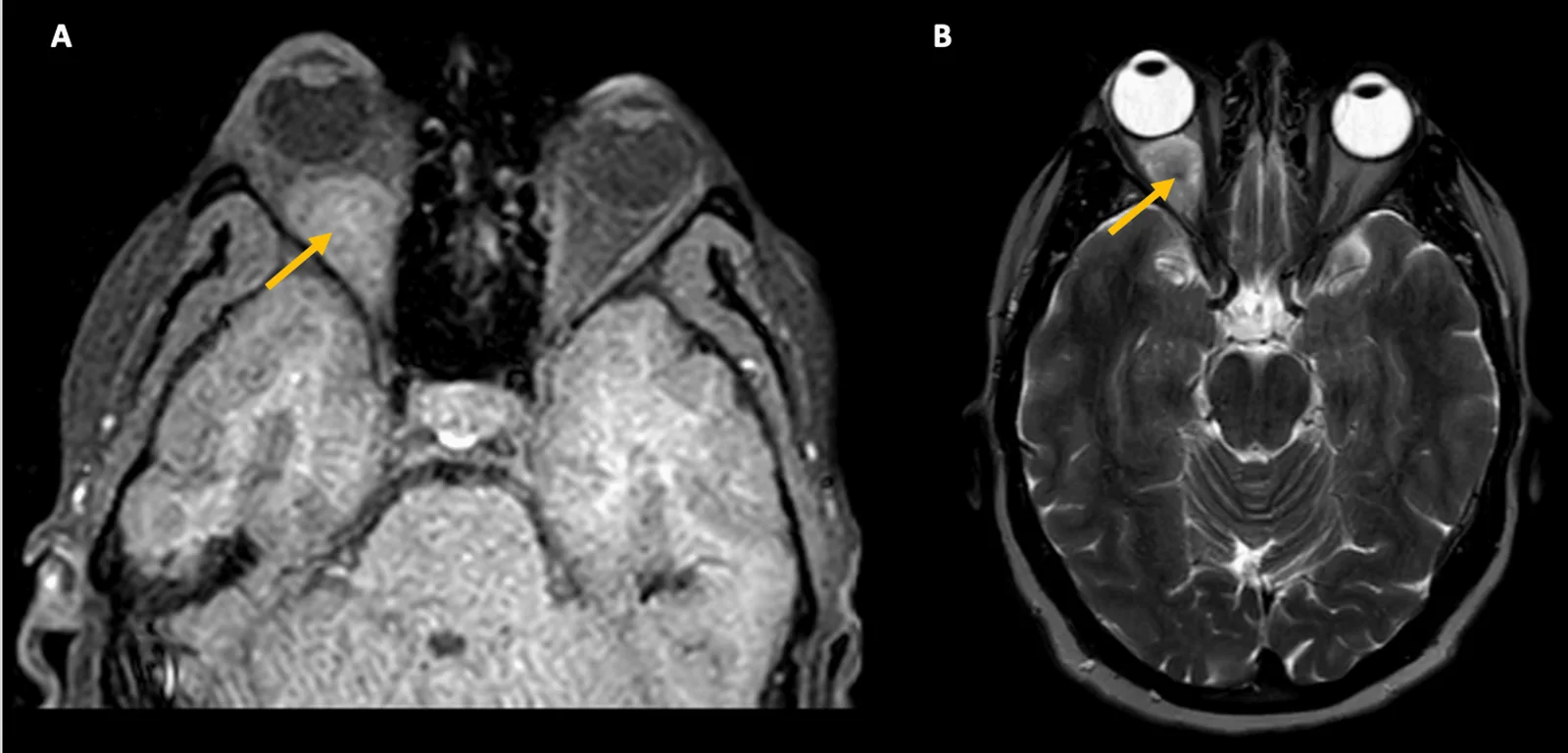
Orbital magnetic resonance imaging confirming the right retrobulbar hematoma Axial orbital magnetic resonance images demonstrating the right intraconal retrobulbar hematoma (yellow arrows). (A) T1-weighted image and (B) T2-weighted image showing the retrobulbar hemorrhagic collection.

Magnetic resonance angiography did not identify an underlying carotid-cavernous fistula, aneurysm, or arteriovenous malformation. The differential diagnosis included OCS secondary to retrobulbar hemorrhage and an underlying orbital vascular abnormality, including carotid-cavernous fistula or arteriovenous malformation; the imaging findings supported retrobulbar hematoma, while magnetic resonance angiography excluded a major vascular abnormality.

Given the severity of the presentation, urgent orbital decompression was indicated. Although bedside lateral canthotomy and cantholysis were considered, the immediate availability of the maxillofacial surgery team and operating room allowed urgent formal surgical orbital decompression to be planned as the primary decompressive strategy. During preoperative management, a single intravenous bolus of methylprednisolone 120 mg was administered. Cardiology consultation also led to resumption and adjustment of antihypertensive treatment with initiation of a fixed-dose combination of valsartan 160 mg and amlodipine 5 mg once daily.

During the preoperative period, after initiation of these medical treatments, the patient developed rapid and sustained clinical improvement. Best-corrected visual acuity in the right eye improved to 10/10 (20/20), intraocular pressure decreased to 18 mmHg, proptosis regressed, and ocular elevation returned to normal. The mild corneal edema also resolved during this clinical improvement. Because the clinical findings that had prompted emergency decompression were resolving before surgery and there was no persistent or progressive evidence of visual compromise, the surgical team deferred decompression in favor of strict ophthalmic observation, with immediate surgical intervention available in the event of deterioration. Following the intravenous methylprednisolone bolus, treatment was transitioned to oral prednisolone 60 mg daily, followed by progressive tapering.

At 24 hours, the improvement was sustained, with best-corrected visual acuity remaining 10/10 (20/20) in the right eye. Automated 24-2 perimetry was within normal limits in both eyes. Repeat orbital CT demonstrated regression of the retrobulbar hematoma, with decreased stranding of the intra- and extraconal orbital fat and marked reduction of the associated proptosis (Figure 6).

**Figure 6:**
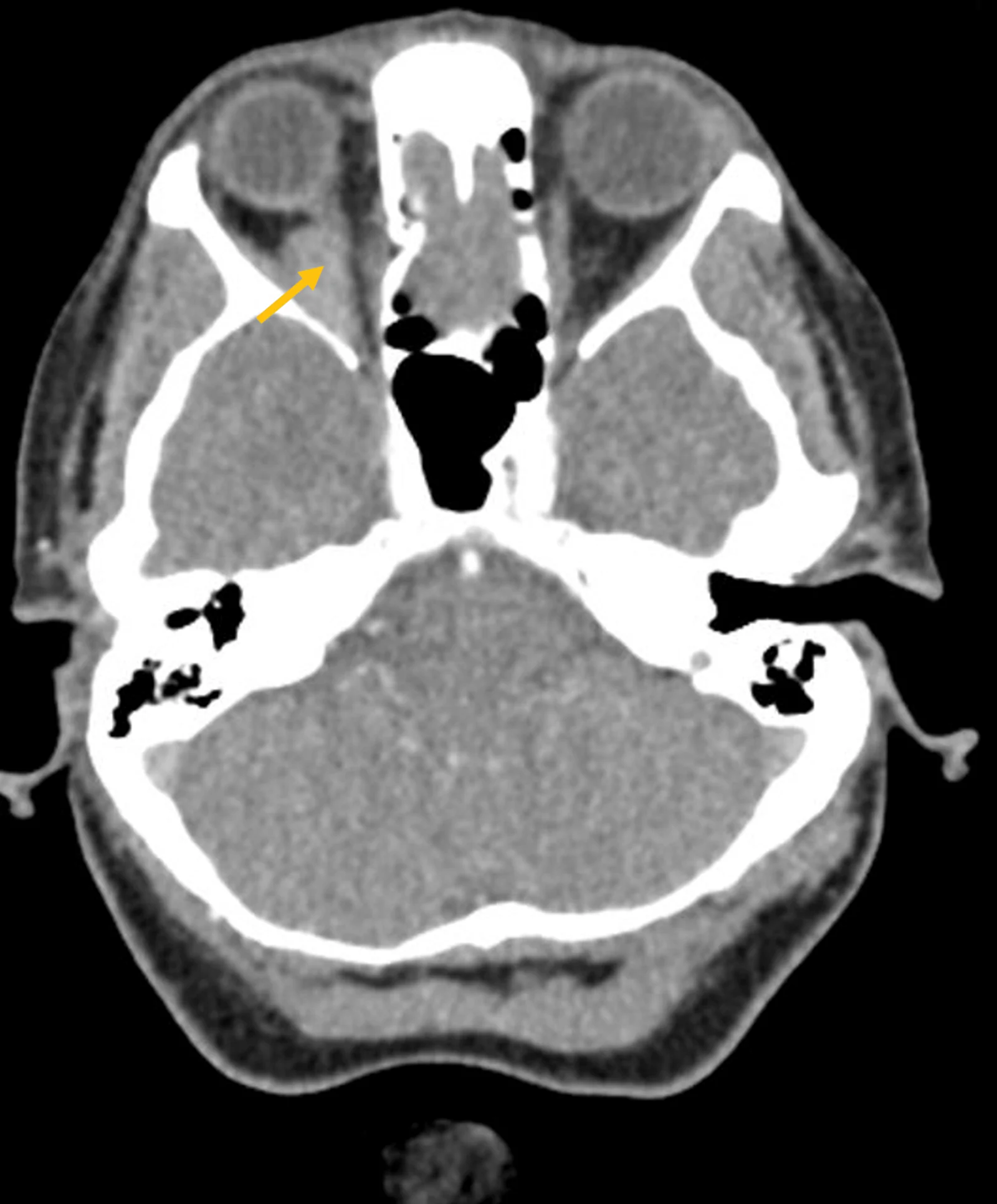
Orbital computed tomography at 24-hour follow-up Axial orbital computed tomography performed 24 hours after presentation showing regression of the right intraconal retrobulbar hematoma (yellow arrow), with decreased stranding of the intra- and extraconal orbital fat and marked reduction of the associated right-sided proptosis.

The patient remained under ophthalmic surveillance and continued parallel cardiology follow-up for blood pressure control.

At four weeks, best-corrected visual acuity in the right eye remained 10/10 (20/20), intraocular pressure was normal, ocular motility was full, and no clinically evident proptosis remained (Figure 7).

**Figure 7:**
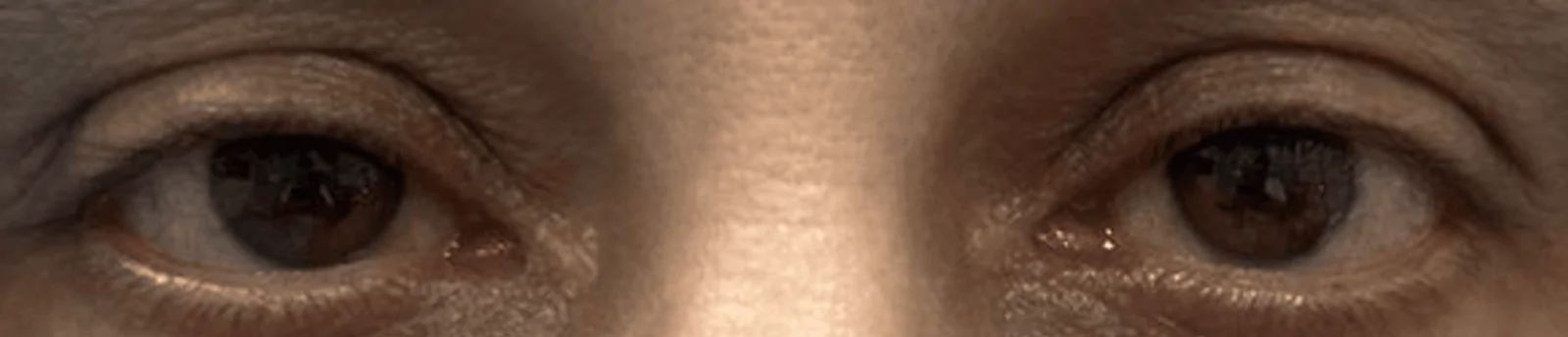
Clinical appearance after resolution Clinical photograph at four-week follow-up showing restoration of symmetric ocular appearance with no clinically evident right-sided proptosis. Written informed consent was obtained from the patient for publication of this case report and accompanying images.

Fundus examination showed complete resolution of the previously noted flame-shaped retinal hemorrhage (Figure 8).

**Figure 8:**
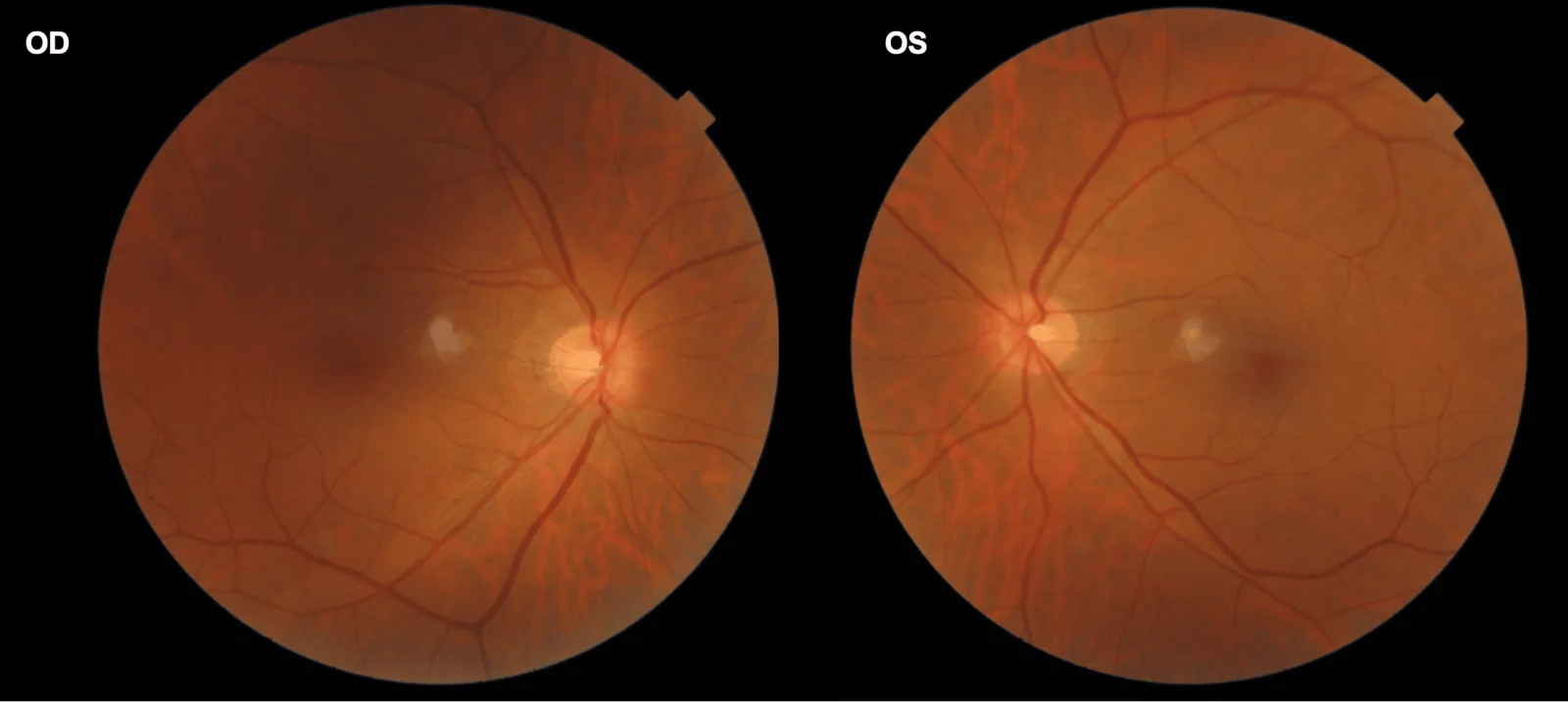
Color fundus photography at four-week follow-up Color fundus photographs obtained at four weeks showing complete resolution of the previously observed flame-shaped retinal hemorrhage in the right eye (OD). The left eye (OS) remained unremarkable.

Follow-up CT confirmed complete radiological resolution of the retrobulbar hematoma (Figure 9).

**Figure 9:**
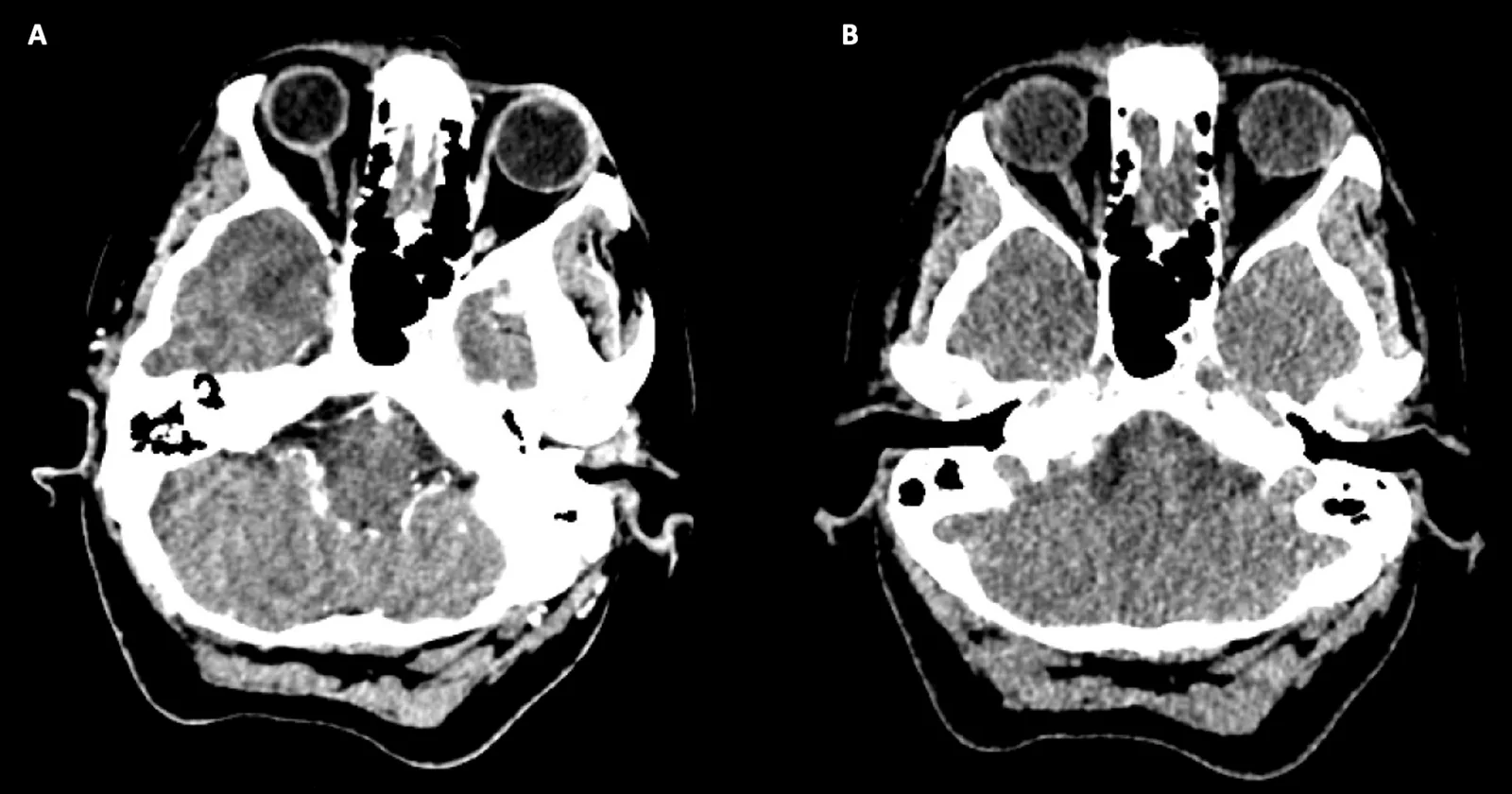
Orbital computed tomography at four-week follow-up Axial orbital computed tomography obtained at four weeks showing complete radiological resolution of the previously observed right intraconal retrobulbar hematoma. (A) and (B) demonstrate the absence of residual retrobulbar hemorrhagic collection and resolution of the associated orbital abnormalities.

## Discussion

Spontaneous retrobulbar hematoma is rare, and its management depends on both the underlying etiology and the degree of orbital pressure-related visual compromise. McNab classified nontraumatic orbital hemorrhage according to anatomic pattern and underlying cause, emphasizing vascular lesions, increased venous pressure, bleeding disorders, inflammatory or infectious disease, and orbital lesions as major categories [3]. In a series of 115 patients with nontraumatic orbital hemorrhage, Sullivan and Wright found an associated orbital vascular malformation in 90%, while additional or alternative predisposing factors included hypertension and coagulopathy [4]. Uncontrolled hypertension has also been described in individual reports of spontaneous retrobulbar hemorrhage [6], and vascular risk factors, including hypertension, have been identified in spontaneous hemorrhage related to the extraocular muscles [7]. In the present patient, the absence of trauma, normal hematologic and coagulation studies, and negative vascular imaging make severe uncontrolled hypertension a plausible systemic association; however, causality cannot be established from a single case.

The initial presentation was highly concerning for OCS. OCS is a clinical emergency characterized by an acute increase in intraorbital pressure and may present with proptosis, a tense orbit, elevated intraocular pressure, visual loss, afferent pupillary dysfunction, dyschromatopsia, and restricted extraocular movements [1,2,8]. Imaging can demonstrate the underlying hemorrhage and provide additional information regarding orbital anatomy, but it remains adjunctive and should not delay decompression when persistent or progressive visual compromise is present [8,9]. In our patient, profound visual loss, marked orbital tension, motility restriction, and a 3.6-mm interocular asymmetry in anterior globe projection on CT, supporting right-sided proptosis, appropriately prompted the initial decision for urgent decompression.

The mechanism of the profound initial visual loss deserves particular consideration. Despite counting-fingers visual acuity, there was no relative afferent pupillary defect, color vision was preserved on the Lanthony desaturated D-15 hue test, and optical coherence tomography with ganglion cell analysis showed no significant structural abnormality. Mild corneal edema was present and may have contributed to the reduction in visual acuity, although its limited severity was unlikely to fully account for such profound visual loss. Conversely, the absence of a relative afferent pupillary defect and preserved color vision make established severe compressive optic neuropathy less likely. The most cautious interpretation is therefore that the acute rise in orbital pressure may have resulted in a transient, reversible visual disturbance, potentially compounded by mild corneal edema, rather than representing established compressive optic neuropathy. The rapid recovery of visual acuity and subsequently normal automated perimetry further support the absence of persistent afferent visual pathway dysfunction.

The possible contribution of medical treatment should also be acknowledged. During preoperative preparation, the patient received intravenous methylprednisolone 120 mg, followed by oral prednisolone 60 mg daily with progressive tapering, while antihypertensive therapy was initiated concurrently. Clinical improvement occurred after these treatments had been started, including resolution of the mild corneal edema, recovery of visual acuity, reduction of intraocular pressure, regression of proptosis, and normalization of ocular motility. A contribution of corticosteroid therapy to this improvement cannot be excluded. However, there was no objective evidence of optic nerve edema, and corticosteroid therapy and blood pressure control were initiated within the same time frame as the observed clinical improvement. The relative contribution of each component of medical treatment therefore cannot be determined from this single observation.

Urgent lateral canthotomy and cantholysis remain the standard first-line decompressive procedure when OCS with visual compromise is suspected [1,2,8]. In our patient, bedside lateral canthotomy and cantholysis were considered, while the immediate availability of the maxillofacial surgery team and operating room allowed urgent formal orbital decompression to be planned. Importantly, the subsequent decision not to proceed with surgery did not represent an initial conservative management strategy. Decompression was deferred only after rapid and sustained objective improvement occurred during the preoperative period, with recovery of visual acuity to 10/10 (20/20), reduction of intraocular pressure to 18 mmHg, regression of proptosis, and normalization of ocular motility, while immediate surgical capability remained available. Real-world data also show that initial decompression is not uniformly successful, reinforcing the importance of serial reassessment of pressure and visual function after intervention [10].

Rare reports have documented visual recovery without decompression in apparent OCS, illustrating that the clinical course is not uniform [11]. More broadly, selected nontraumatic orbital hemorrhages may be managed without surgery when visual function remains preserved or improves, although operative intervention is required when visual compromise persists or progresses [3,4]. Conservative management has also been described in selected spontaneous retrobulbar hematomas with preserved or improving visual function [12]. Conversely, a recent 2026 report described a spontaneous retrobulbar hematoma occurring during a hypertensive emergency that ultimately required supraorbital craniotomy, illustrating the heterogeneity of clinical course and the need to tailor management to visual function, hematoma evolution, and anatomic characteristics [13]. These observations support careful individualized reassessment but do not alter the principle that persistent or progressive visual compromise requires urgent decompression.

In the present case, repeat CT at 24 hours demonstrated interval regression of the hematoma, while automated 24-2 perimetry performed at 24 hours was within normal limits in both eyes. At four weeks, visual acuity remained 10/10 (20/20), intraocular pressure was normal, ocular motility was full, and no clinically evident proptosis remained. Fundus examination showed complete resolution of the previously observed flame-shaped retinal hemorrhage, while follow-up CT confirmed complete radiological resolution of the retrobulbar hematoma.

This report has several limitations. Initial intraocular pressure could not be quantified because of extreme globe firmness, and standardized baseline Hertel exophthalmometry and nine-gaze photography were not obtained during the emergency assessment. The precise mechanism of the initial profound visual loss could not be established with certainty, and the respective contributions of corticosteroid therapy and blood pressure control to the observed recovery cannot be separated in a single case. Nevertheless, serial ophthalmic examinations, functional testing, and multimodal orbital imaging provided objective documentation of the clinical and radiological course.

## Conclusions

Spontaneous retrobulbar hematoma should be considered in patients presenting with acute painful unilateral proptosis, even in the absence of trauma. Severe uncontrolled hypertension may represent an associated systemic factor, although a causal relationship cannot be established from a single case. When OCS is suspected, the priority remains rapid assessment of visual function and urgent decompression when visual compromise persists or progresses.

In this patient, urgent surgical decompression was initially planned because of profound visual loss, marked orbital tension, proptosis, and motility restriction. Rapid and sustained objective improvement occurred during preoperative management after initiation of corticosteroid and antihypertensive therapy, allowing surgery to be deferred under strict ophthalmic monitoring with immediate surgical capability available if deterioration occurred. The respective contributions of corticosteroid therapy and blood pressure control to the observed recovery cannot be determined. This case emphasizes the value of repeated objective reassessment when the clinical course changes, without suggesting that observation should replace urgent decompression in patients with persistent or progressive visual compromise.
